# In-depth validation of total HIV-1 DNA assays for quantification of various HIV-1 subtypes

**DOI:** 10.1038/s41598-018-35403-6

**Published:** 2018-11-22

**Authors:** Sofie Rutsaert, Ward De Spiegelaere, Clarissa Van Hecke, Marie-Angélique De Scheerder, Maja Kiselinova, Karen Vervisch, Wim Trypsteen, Linos Vandekerckhove

**Affiliations:** 1 HIV Cure Research Center, Department of Internal Medicine, Ghent University, Ghent, Belgium; 2 Department of Morphology, Faculty of Veterinary Medicine, Ghent University, Ghent, Belgium; 3 Department of Internal Medicine, Ghent University, Ghent, Belgium

## Abstract

HIV-1 DNA quantification serves as an important reservoir biomarker in HIV cure trials. However, the high genetic diversity of HIV-1 represented by different subtypes may bring inaccuracy in quantifying HIV-1 DNA and a sensitive and validated assay covering diverse HIV-1 subtypes is lacking. Therefore, we cross-validated total HIV-1 DNA assays described in literature using a three-step comparative analysis. First, a bioinformatics tool was developed in-house to perform an *in silico* evaluation of 67 HIV-1 DNA assays. Secondly, these selected assays were *in vitro* validated using a panel of different HIV-1 subtypes and, finally, *ex vivo* assessed on selected patient samples with different HIV-1 subtypes. Our results show that quantification of HIV-1 DNA substantially differs between assays and we advise five best performing HIV-1 DNA assays for ddPCR and qPCR (Schvachsa_2007, Viard_2004, Heeregrave_2009, Van_der_Sluis_2013, Yu_2008 and Yun_2002). This in-depth analysis of published HIV-1 DNA assays indicates that not all assays guarantee an optimal measurement of HIV-1 DNA, especially when looking across subtypes. Using an in-depth cross-validation, we were able to validate HIV-1 DNA assays that are suitable for quantification of HIV-1 DNA in a wide variety of HIV-1 infected patients.

## Introduction

Total HIV-1 DNA in HIV-1 infected patients is increasingly being recognized as a valuable marker for HIV cure efforts, but also for current clinical follow-up. Upon infection, HIV-1 integrates into the human genome where it can remain dormant as proviral DNA in infected cells and thereby avoiding eradication by antiretroviral therapy (ART). This impedes an HIV cure because the reservoir of latently infected cells can fuel viral rebound when ART is interrupted^1^. To date, different HIV cure strategies are being explored in order to reduce or limit the establishment of the viral reservoir, and in this context, total HIV-1 DNA is increasingly being used as a marker for the size of the viral reservoir^2,3^. Furthermore, the role of HIV-1 DNA quantification is also being explored in treatment simplification strategies as virological control under monotherapy has been correlated to a lower level of total HIV-1 DNA compared to patients who failed protease inhibitor monotherapy^3–5^. This suggests that total HIV-1 DNA is a promising predictive factor for treatment stratification in HIV-1 infected patients. Lastly, precise HIV-1 DNA quantification is essential for early HIV diagnosis of infants born to HIV-positive morthers^6^. As illustrated, total HIV-1 DNA is established as a relevant marker but inaccuracy in the quantification can arise from the high genetic heterogeneity of HIV-1 that is reflected by the various HIV-1 strains and subtypes among patients and the high variation of sequences within patients. In Western countries the HIV-1 subtype B is dominant and many described HIV-1 DNA assays are focused on the quantification of this subtype^7^. However, more detailed knowledge about their performance in quantifying HIV-1 DNA across all HIV-1 subtypes is necessary in order to contribute to HIV cure trials worldwide. For that reason, there is a growing need to invest in identifying a uniform total HIV-1 DNA assay that is able to accurately quantify different HIV-1 subtypes to be used in a global clinical setting. Here, we present a three-step comparative analysis of already published HIV-1 DNA PCR assays. First, a bioinformatics tool was designed to compare HIV-1 DNA assays described in literature, subsequently the assays were further assessed on cells infected with panel of HIV-1 viruses, and lastly a comparison was made using relevant patient material from individuals infected with various HIV-1 subtypes.

## Results

### Selection of total HIV-1 DNA assays and in silico prediction analysis

**Figure 1:**
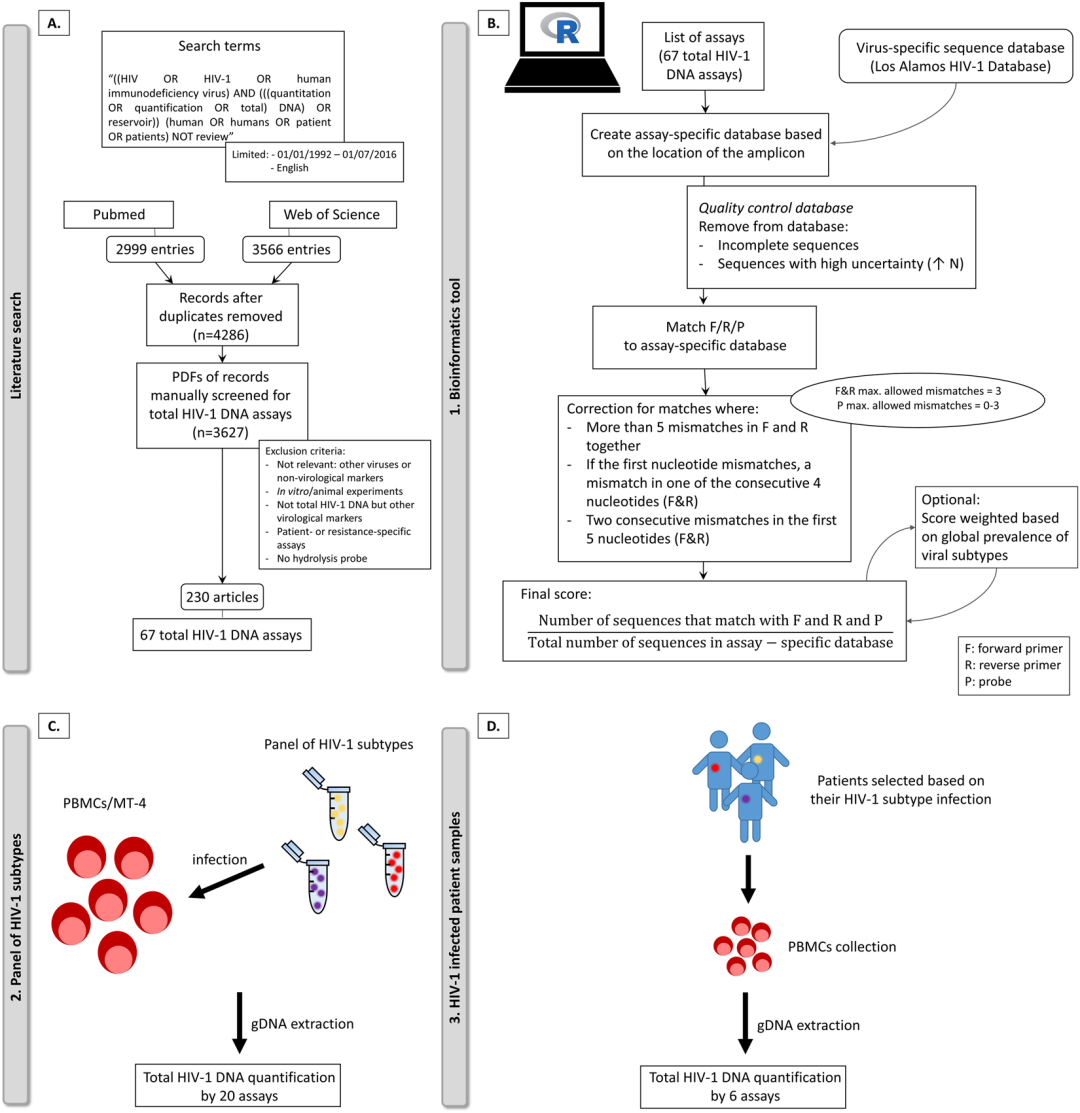
Schematic representation of workflow with (**A**) the literature search, (**B**) an in-house developed bioinformatics analysis pipeline and analysis of HIV-1 DNA assays on (**C**) a panel of HIV-1 subtypes and (**D**) HIV-1 infected patient samples. F: forward primer, R: reverse primer, P: probe, N: any nucleotide, PBMCs: peripheral blood mononuclear cells.

**Figure 2:**
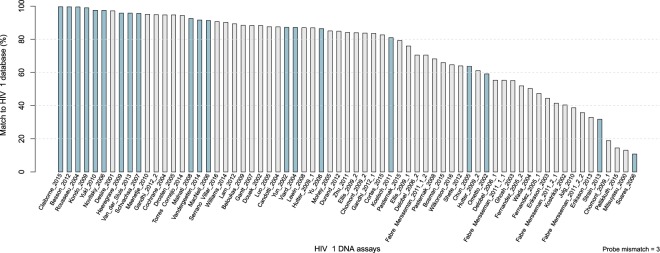
In silico evaluation of total HIV-1 DNA assays. HIV-1 DNA assays are ranked based on the percentage of matching to HIV-1 Los Alamos database (blue bars represent the HIV-1 DNA assays selected for further *in vitro* analysis).

A total of 3627 articles were scanned in search of PCR-based assays used for quantification of total HIV-1 DNA in patients (Fig. 1A). As a result, 67 HIV-1 DNA assays were analyzed *in silico* by using the in-house developed bioinformatics tool (Fig. 1B, Supplemental Table S1). The 5 top ranked assays were selected and complemented with additional 13 HIV-1 DNA assays that were frequently cited (≥6 citations) in literature (Fig. 2). In addition, two assays were included for the validation of the tool, i.e. Novitsky_2006, due to a substantial impact of the number of allowed mismatches for the probe on its ranking (Supplemental Fig. S1) and Soares_2006 due to a low ranking based on the bioinformatics tool. In total, 20 HIV-1 DNA assays were selected for further evaluation.

### Performance of HIV-1 DNA assays on a panel of HIV-1 subtypes by ddPCR

**Figure 3:**
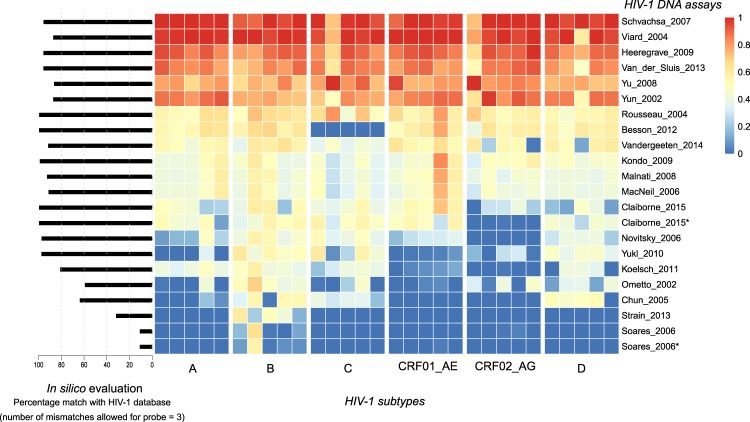
Validation of a panel of HIV-1 subtypes. Heatmap of HIV-1 DNA assays analyzed on DNA of a panel of HIV-1 patient isolates of different HIV-1 subtypes by ddPCR. Relative HIV-1 DNA quantification results per sample show the assays with high sensitivity (red) and the assays with lower sensitivity (blue). (*) DNA samples restricted with restriction enzyme XhoI instead of EcoRI prior ddPCR quantification.

In total, 30 samples were analyzed, five per HIV-1 subtype (HIV-1 A, B, C, D, CRF01_AE and CRF02_AG) by the 20 selected assays (Fig. 1C, Fig. 3). The capability to quantify HIV-1 DNA of different subtypes varied greatly between assays. In agreement with the *in silico* data, Soares_2006 only picked up minimal HIV-1 DNA sequences from various subtypes. Multiple assays such as Koelsch_2011, Yukl_2010, Novitsky_2006 were able to amplify HIV-1 subtype B, but underperformed with other subtypes, in particular HIV-1 subtypes CRF01_AE and CRF02_AG. Besson_2012 was able to quantify HIV-1 DNA efficiently but was unsuccessful in capturing HIV-1 DNA from subtype C. Six assays (Schvachsa_2007, Viard_2004, Heeregrave_2009, Van_der_Sluis_2013, Yu_2008 and Yun_2002) were able to capture more than 70% of the HIV-1 DNA in >90% of the samples with varying HIV-1 subtypes. Two out of these six (Schvachsa_2007 and Viard_2004) were able to capture more than 90% of HIV-1 DNA in >90% of the samples across all HIV-1 subtypes.

### HIV-1 DNA assays in patient samples (ddPCR and qPCR)

**Figure 4:**
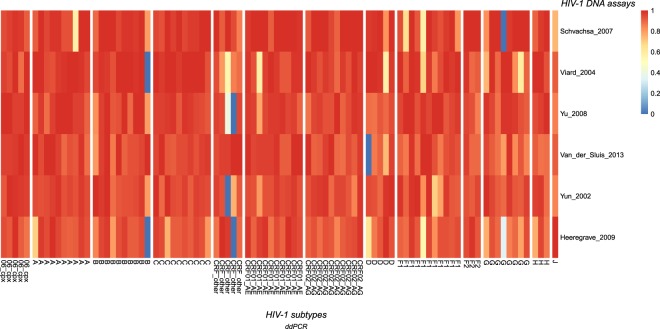
Validation on patient samples selected based on their HIV-1 subtype infection. Heatmap of HIV-1 DNA assays analyzed on DNA of HIV-1 infected patients by ddPCR (log_10_ HIV DNA/10^6^ PBMCs). Relative HIV-1 DNA quantification results per patient show the assays with high sensitivity (red) and the assays with lower sensitivity (blue).

The six best assays were selected for subsequent in-depth analysis in HIV-1 infected patient samples. Here, HIV-1 DNA was quantified by ddPCR in 91 HIV-1 positive patients, categorized in thirteen different HIV-1 subtypes (Table 1, Fig. 1D, Fig. 4, Supplemental Table S2). All assays were able to capture on average 90% of the maximally measured HIV-1 DNA (Schvachsa_2007: 94.2%, Viard_2004: 93.0%, Yu_2008: 93.3%, Van_der_Sluis_2013: 92.8%, Yun_2002: 92.7% and Heeregrave_2009: 88.5%). Additionally, the performance of these assays were analyzed on the qPCR platform in 87 patient samples (Table 1, Fig. S2, Supplemental Table S3). Correspondingly, the HIV-1 DNA assays were able to amplify around 90% of the maximally measured HIV-1 DNA (Schvachsa_2007: 90.0%, Viard_2004: 93.3%, Yu_2008: 93.7%, Van_der_Sluis_2013: 88.5%, and Yun_2002: 91.4%) except Heeregrave_2009 which had an average of 73.5%.

**Table 1:** Validation of 6 selected HIV-1 assays on patient samples selected based on their HIV-1 subtype infection.

HIV-1 subtypes	Number of patients included	Number of patients included
patients	ddPCR	qPCR
B	10	10
CRF02_AG	10	10
CRF01_AE	10	10
A	10	9
C	10	10
F1	11	10
G	8	6
CRF_other	5	5
D	5	5
06_cpx	5	5
H	3	3
F2	3	3
J	1	1
Total number of patients	91	87

Number of patients per HIV-1 subtype is mentioned based on sample availability (HIV-1 subtype 06_cpx stands for an HIV-1 A/G/J recombinant).

### ddPCR considerations: restriction enzymes and LTR-region of HIV-1 genome

As restriction enzymes are often used in ddPCR workflows to ensure gDNA digestion and proper encapsulation of gDNA into droplets, commonly used restriction enzymes were evaluated on HIV-1 DNA fragmentation by *in silico* predictions of target cut sites within the HIV-1 genome (Supplemental Fig. S2). EcoRI was selected for enzymatic restriction of samples for all HIV-1 DNA assays. However, EcoRI cuts in the amplicon of Assays Claiborne_2015 and Soares_2006 in respectively 33.54% and 6.46% of the HIV-1 sequences of the Los Alamos database (Supplemental Fig. S3). Therefore, additional samples were restricted with restriction enzyme XhoI for the quantification by the two above mentioned assays. However, no substantial difference in HIV-1 DNA quantification was observed by the selection of the restriction enzymes for those two assays (Fig. 3). Furthermore, the target amplicon of assay Vandergeeten_2014 and Chun_2005 is located solely in the LTR-region of HIV-1 genome. Due to the two LTR regions present in the HIV-1 genome, these assays measure up to a twofold overestimation of number of HIV-1 DNA copies (Supplemental Fig. S4). Therefore, for these assays a correction factor of 1/2 was applied.

## Discussion

A validated HIV-1 DNA assay that can reliably and accurately quantify different HIV-1 strains in patient derived samples is a prerequisite for the applicability of total HIV-1 DNA as a biomarker of HIV-1 reservoir in clinical care and experimental HIV cure trials. Various PCR-based total HIV-1 DNA assays and approaches have been described in literature^8–12^. However, a comprehensive comparative analysis of the published assays has not been done yet. Our literature search indicated that more than 60 different assays for quantifying total HIV-1 DNA are being used in studies with patient samples in the last 2 decades. This raises the concern about comparing quantitative data between different laboratories.

Since a wet-lab validation of this large amount of assays is costly, we opted to implement a data analysis pipeline for the initial *in silico* comparison of assays. Our results show that this tool provides a rough estimate of the true performance of the assays. But nonetheless, a subsequent wet-lab validation of assays remains the essential step of the validation process. The strength of the tool lies in the comprehensive analysis of primers and probes together and the ability to allow a limited number of primer and probe mismatches. At present, too little data is available to quantitatively assess the assay performance on specific mismatches as the effect of mismatches depends on the specific base-pairs^13^, the neighboring base pairs^14,15^ and the position on the primer or probe^14,15^. Future studies aiming to quantitatively assess this issue may enable a further fine-tuning of our pipeline. Additionally, this type of data analysis pipeline is not limited only to the HIV field. It is therefore designed in an open-source platform and allows for the analysis of assays of other viruses or organisms with high variable genomes.

The initial wet-lab validation of the panel of 5 different HIV-1 subtypes indicated that there are large differences in total HIV-1 DNA quantification between these assays. This hampers data comparison between results from different studies and may result in biased conclusions. It is important to note here that some assays were intended for a specific subtype and/or set of patients^16–19^, whereas other assays were validated on multiple subtypes^20^. However, implementation of these assays in patients with unknown subtypes of different subtypes of the ones initially designed for may lead to misleading data where results will most probably underestimate HIV-1 quantification. A pan-HIV-1 assay is preferable since HIV-1 subtypes are not always known, and pan-HIV-1 assays are less refractory to variability within subtypes or patients.

Accurate HIV-1 DNA quantification of patient samples is highly challenging because of the low abundance of HIV-1 DNA in patients suppressed with ART and the viral heterogeneity within this patients. Therefore, the six selected assays were tested on patient samples, selected based on their HIV-1 subtype, by ddPCR and qPCR. Data generated from the HIV-1 infected patients confirm the data from the HIV-1 panel. On ddPCR, all assays where able to pick up around 90% of the percentage of HIV-1 DNA copies in relation to the assay with the most detected copies (88.63–94.25%) and have a similar performance. Some samples have a relative value of 0 (blue in Fig. 4 and Supplemental Fig. S5) but these can be explained by a general low concentration of the HIV-1 DNA present in that particular sample indicating that the effect of sampling variation may have caused these results^21^. The adequate performance from these assays was confirmed by data generated on the qPCR platform. However, for qPCR the performance of Heeregrave_2009 drops to an average of 72.9%. For the qPCR platform, primer/probe mismatches can cause detrimental effect on PCR efficiency and lead to an underestimation of the true HIV-1 DNA level^13^. For ddPCR, mismatches between primer/probe and target DNA or inhibitory substances cause a drop in the fluorescence of the droplets and leads to droplets with an intermediate fluorescence^22,23^. But these droplets with intermediate fluorescence can remain quantifiable in the ddPCR^22^.

There are some limitations associated with this study. In the literature search, some assays may have been missed due to a non-availability of full-texts or assays published after July 2016. Furthermore, the bioinformatics tool has its restrictions. A number of mismatches of the probe still permits efficient amplification, but only minimal information is available on the influence of the location of these mismatches, which lead to the simplification of the tool where a varying 0 to 3 mismatches were allowed for the probe. Various primers and/or probe had degenerative nucleotides, this could be considered as a mismatch in disguise and could skew the ranking. Additionally, the performance of the tool depends on the quality of database: for some HIV-1 subtype only a few representative sequences were available and the 3′end of the sequences in the HIV-1 database is often truncated: assays located in the 3′ end of the HIV-1 genome (3′-LTR) were only assessed on a fifth of the total amount of sequences in the HIV-1 database. However, the bioinformatics tool allows to use a viral database of choice.

Overall, this study highlights the limitation of comparing results between studies using different assays for HIV DNA quantification and the importance of identifying a validated HIV-1 DNA assay that accurately measures total HIV-1 DNA for its further implementation in HIV-1 clinical trials. Since PCR-based HIV DNA quantification has a good inter-laboratory reproducibility^3,24^, a validated assay will allow the comparison of results between different clinical studies and different laboratories but good standards will be necessary for validation of the method, especially for qPCR which requires an stable external calibrator^25^. Lastly, a precise quantification of HIV-1 DNA can guide the selection of patients participants in HIV-1 cure studies and early diagnosis of infants of seropositive mothers. We do want to point out that while digital PCR is a good quantification method for HIV DNA load, it is not an optimal technique for early diagnosis of HIV since low level of false positive droplets cannot be excluded^26^. Furthermore, the 5′-LTR and 3′-LTR within one HIV genome can be separated due to fragmentation or restriction digest, and incorporated into two different droplets. Thereafter, HIV DNA quantification could lead to a twofold overestimation if the used assay is located exclusively in the LTR region. Ample amount of assays are described in literature, but to our knowledge, a cross-validation of assays has not been performed before. This type of validation enables expansion of cohorts to patients infected with non-B HIV-1 subtype, and include a varied group of patients with different background and HIV-1 subtypes.

Finally, we hereby declare that the study was performed without any personal or financial bias towards any described assay.

## Materials and Methods

### Systematic literature search

To obtain a comprehensive list of frequently used HIV-1 DNA assays, a systematic literature screening was performed using PubMed and Web of Science databases (Fig. 1A). Articles were included that describe quantification of HIV-1 DNA, total HIV-1 DNA and HIV-1 reservoir in the context of patient samples for the period between 1^st^ of January 1992 and 1^st^ of July 2016. The following search terms were used: (HIV OR HIV-1 OR human immunodeficiency virus) AND (((quantitation OR quantification OR total) DNA) OR reservoir)) (human OR humans OR patient OR patients). The inquiry was further refined by excluding reviews and limited to articles written in English. All remaining articles were pooled and duplicates were removed. Subsequently, availability of the full-text of the articles was checked which led to a database of 3627 articles. This set of articles was manually evaluated with following exclusion criteria: (1) quantification of non-virological markers or other viruses than HIV-1; (2) quantification in non-patient samples such as *in vitro* or animal experiments; (3) quantification of other virological markers such as long terminal repeat (LTR) circles, HIV-1 RNA, integrated HIV-1 DNA; (4) patient-specific or resistance-related assays; (5) total HIV-1 DNA assays lacking a hydrolysis probe and (6) total HIV-1 DNA assays with a publicly unavailable sequence. Since we aimed at validating assays that are compatible for the most commonly used platforms: droplet digital PCR (ddPCR) and real-time PCR (qPCR), (7) assays using the fluorescence resonance energy transfer technology were further discarded. For further validation of nested PCR approaches, the inner primer pair and probe were selected and assays consisting of multiple primers/probes in one reaction were divided in single sets of primers (forward and reverse) and a single probe.

### In silico analysis of HIV-1 DNA assays using an in-house designed bioinformatics tool

To analyze the theoretical performance of the assay, a bioinformatics analysis pipeline was developed in R language (Fig. 1B, script available upon request)^27^. In this bioinformatics tool, assays consisting of a set of primers and a probe are matched to a database of HIV-1 sequences. For our analysis, a comprehensive database of HIV-1 sequences was extracted from the HIV Los Alamos National Laboratory website (www.hiv.lanl.gov)^28^. Since the database is incomplete in some HIV-1 DNA regions, assay-specific databases were constructed based on the subsampling of sequences that had a complete sequence annotation in the region of the primers and probes. Alignment of matched primers and probes was performed on the assay-specific database, and hits were only recorded when both the primers and probe matched to a particular sequence. To enable comparison of different assays, the total number of hits was divided by the total number of sequences in the assay specific database. As PCR is to some extent refractory to primer/probe mismatches, the tool can allow a certain number of mismatches. Here, up to maximum 3 mismatches for the primers and a varying maximum 0, 1, 2 or 3 mismatches for probe was allowed. However, we corrected for specific deleterious mismatches that are likely to block the reaction completely^14,15^: (1) five or more mismatches in the forward and reverse primer combined; (2) mismatch in the first 3′-end nucleotide when additional mismatches occurred in one of the four consecutive nucleotides and (3) two consecutive mismatches in the first five 3′-end nucleotides in the primers were not allowed. Furthermore, the score was weighted based on the global prevalence of the different HIV-1 subtypes to correct for misrepresentation of specific subtypes in the database (e.g. overrepresentation of HIV-1 subtype B)^29^. Sequences of primers and probes of all assays are summarized in Supplemental Table S1.

### Assay optimization for droplet digital PCR (ddPCR) and qPCR

Based on the bioinformatics tool and complemented with assays frequently described in literature, a selection of 20 HIV-1 DNA assays was chosen to perform wet-lab validation (Fig. 1C,D). PCR protocols were optimized for each of the selected HIV-1 DNA assays on the droplet digital PCR QX200 (Bio-Rad) and qPCR LightCycler® 480 (Roche Applied Science) by performing a temperature gradient PCR to select assay-specific annealing/elongation temperatures (Supplemental Table S1).

### Comparison of selected HIV-1 DNA assays on a standardized HIV-1 subtype panel

#### Virus stocks, cell culture, infection and sample processing

An initial validation was performed by ddPCR on a panel of HIV-1 viruses obtained from the National Institutes of Health (NIH) (Panel of 60 International HIV-1 Isolates, Cat# 11412,^30^). This panel consists of viral HIV-1 isolates of persons infected by subtype A, B, C, D and circulating recombinant forms (CRF), CRF01_AE and CRF02_AG. To generate DNA sequences *in vitro*, infection was performed on peripheral blood mononuclear cells (PBMCs) from healthy donors or MT-4 depending on the HIV-1 tropism (R5 or X4) of the viral isolates. Briefly, 200 µl of virus was added to 3 × 10^6^ cells and after spinoculation (900 g, 90 min, 32 °C) kept in culture at 5% CO_2_ at 37 °C. After 7 days of culture, cells were harvested and stored as a dry-pellet of 10^7^ at −80 °C. To confirm successful infection, an aliquot of supernatant was assessed by the Retroviral Reverse Transcriptase (RT) assay^31^.

#### Total HIV-1 DNA quantification by droplet digital PCR

Five distinct viruses per HIV-1 subtype (HIV-1 A, B, C, D, CRF01_AE and CRF02_AG) were analyzed by ddPCR (total of 30 samples). Total genomic DNA (gDNA) was isolated from 10^7^ cells using DNeasy Blood & Tissue Kit (Qiagen) according to manufacturer’s protocol. DNA was eluted in 100 µl elution buffer and kept at 56 °C for 10 min to maximize the DNA yield. Next, gDNA was restricted by EcoRI or XhoI, (restriction enzyme selection described in Supplement; EcoRI for all assays, extra samples restricted by XhoI for Claiborne_2015 and Soares_2006) with the use of 12.98 µl gDNA in a total volume of 15 µl of restriction mix. The ddPCR mix was made by adding 2 µl of sample to 10 µl of 2x ddPCR supermix for probes (Bio-Rad), 500 nM of primers and 300 nM of probe (Integrated DNA Technologies) in a final volume of 20 µl. Samples were measured in duplicate. The PCR protocol consisted of initial denaturation at 95 °C for 10 min, followed by 40 cycles of 95 °C for 30 s denaturation and assay-specific annealing/elongation temperature (Supplemental Table S1) for 60 s with a ramp rate of 2.5 °C/s, and completed by an enzyme deactivation step of 98 °C for 10 min. Droplets were read by the QX200 droplet reader (Bio-Rad) and samples were quantified with the ddpcRquant software^22^. Graphically representation by heatmap was performed with the pheatmap package (version 1.0.8) in R^27^. As we cannot make an absolute estimation of the total HIV-1 DNA per sample, for each sample the concentration of HIV-1 DNA copies from an assay was divided by the output of the assay that quantified the largest amount of HIV-1 DNA within the same sample. This led to a value, called ‘relative quantification’, between 0 and 1: a result diverging to 0 (0%) (depicted in blue in Fig. 2) means that only a few HIV-1 DNA copies were picked up by the assay compared to the most sensitive assay, whereas a number close to 1 (100%) (red in Fig. 2) stands for a well performing reaction. Due to the fact that their target amplicon is solely located in the LTR region of HIV-1, for Vandergeeten_2014 and Chun_2005 a correction factor of 1/2 was applied (Supplemental data).

### Comparison of selected assays on HIV-1 infected patient samples

Based on performance of the HIV-1 DNA assays on the HIV-1 isolates panel, 6 assays were selected for further validation on patient samples by ddPCR and qPCR. After obtaining written letters of informed consent, HIV-1 infected patients were selected and enrolled based on their HIV-1 subtype at the AIDS Reference Center, Ghent University Hospital, Ghent, Belgium. The study was approved by the Ethics Committee of Ghent University Hospital (Reference number: B670201317826) and the methods were carried out in accordance with the relevant guidelines and regulations. From 91 patients (Table 1), blood was drawn in six 9 ml EDTA tubes and PBMCs were isolated using density gradient centrifugation with Lymphoprep (ELITech Group). Aliquots of 10^7^ PBMCs were stored in freezing media (Fetal Calf Serum + 10% DMSO) at −80 °C. Total gDNA was isolated as described earlier and eluted in 75 µl elution buffer. Total HIV-1 DNA was measured by ddPCR as described previously. The reference gene RPP30 was measured to correct for cell input^32^. Normalized HIV-1 DNA copies were expressed as log_10_ HIV DNA/10^6^ PBMCs. Assays were ranked based on the average of the percentage of HIV-1 DNA copies in relation to the assay with the most detected copies. Based on the availability, 87 samples were additionally measured by qPCR (Table 1, described in Supplement).

## Electronic supplementary material

Supplemental Information

Supplemental Table S1

Supplemental Table S2

Supplemental Table S3

## Data Availability

All data generated or analyzed during this study are included or referred to in this published article (and its Supplementary Information files).
