# Supplementary material for: In-depth validation of total HIV-1 DNA assays for quantification of various HIV-1 subtypes
Source: Sci Rep. 2018 Nov 22;8:17274. doi: 10.1038/s41598-018-35403-6 (PMC6250682; doi:10.1038/s41598-018-35403-6)
Supplement: Supplementary file 1 — Supplemental Information [file 41598_2018_35403_MOESM1_ESM.pdf]

SUPPLEMENTAL INFORMATION

Title: In-depth validation of total HIV-1 DNA assays for quantification of various HIV-1 subtypes.

Authors: Sofie Rutsaert[1], Ward De Spiegelaere[2], Clarissa Van Hecke[1], Marie-Angélique De Scheerder[1], Maja Kiselinova[3], Karen Vervisch[1], Wim Trypsteen[1§], Linos Vandekerckhove[1§*]

§ authors contributed equally

Affiliations:

1. HIV Cure Research Center, Department of Internal Medicine, Ghent University, Belgium
2. Department of Morphology, Faculty of Veterinary Medicine, Ghent University, Belgium
3. Department of Internal Medicine, Ghent University, Belgium

Corresponding author (*): Linos Vandekerckhove; Medical Research Building 2, Corneel Heymanslaan 10, 9000 Ghent, Belgium; Tel: +3293323398, Fax: +3293323895; Linos.Vandekerckhove@UGent.be

Supplemental data

Restriction enzymes analysis for ddPCR implementation

Prior to PCR amplification, an enzyme restriction digestion is performed on genomic DNA (gDNA) for the ddPCR platform. This step is preferred, especially at concentrations >66 ng/reaction, as the fragmented DNA will be more uniformly distributed in all droplets compared

to full-length chromosomal DNA, and to improve target accessibility (Bio-Rad Droplet Digital PCR - Application Guide). Therefore, different restriction enzymes were analyzed. New England Biolabs® Inc. (NEB) specifies a list of 34 restriction enzymes that have been successfully used for DNA digestion before ddPCR (NEB - restriction enzymes for droplet digital pcr). However, it is important that the restriction enzyme does not have a recognition site present in the target amplicon and consequently breaks up the amplicon. The recognition sites in the HIV-1 genome (i.e. all available sequences of the HIV Los Alamos database) were analyzed with an in-house analysis pipeline written in the R software environment (R Development Core Team, 2016, script available upon request). The recognition sites of restriction enzymes were mapped to all sequences of the HIV Los Alamos database and the frequency of the occurrence of recognition sites per enzyme is represented in Figure S2. Additionally, we analyzed the occurrence of restriction recognition sites in the specific target amplicon of the 20 selected total HIV-1 DNA assays (Fig. S3). Restriction enzyme EcoRI has been routinely used at our lab. However, EcoRI cuts in the target amplicon of assay Claiborne_2015 (33.54%) and assay Soares_2006 (6.46%) (Fig. S3). To ensure that EcoRI does not influence the HIV-1 DNA quantification, restriction enzyme was selected for additional samples. Restriction enzymes XhoI and NcoI cut in less than 1% of the sequences contained within the HIV-1 Los Alamos database (Fig. S3) where XhoI had the most optimal restriction for ddPCR implementation (data not shown). Therefore, gDNA of both these assays was additional restricted with restriction enzyme XhoI for measurement of HIV-1 DNA by Claiborne_2015 and Soares_2006 on the ddPCR platform.

Correction for quantification by total HIV-1 DNA assays located in the HIV-1 LTR region

When transferring an assay from the qPCR platform to the ddPCR platform, it is essential to consider the location of the amplicon of the assay. ddPCR technology provides absolute

quantification by counting the PCR-positive and PCR-negative droplets. The HIV-1 genome contains two identical LTR regions, one at the 3' end and one at the 5' end. When an HIV-1 genome is broken by restriction or mechanical shearing, the two LTR region can be separated. These two separated LTR sequences can be embedded in two separate droplets and one HIV-1 DNA strand can give rise to two positive droplets. Two HIV-1 DNA assays out of the selected 20 assays are located solely in the LTR region, Chun_2005 and Vandergeeten_2015 ("LTR-assays"). To prove this hypothesis, we extracted DNA from ACH2 HIV-1 latent cell line and HIV-1 NL4.3 infected SupT1 and diluted genomic DNA below 66ng/ddPCR reaction. Extracted DNA was split in two: one sample was handled with care and not restricted whereas the other sample was vortexed vigorously and restricted by EcoRI. HIV-1 DNA was measured by ddPCR as described before by assay Chun_2005, Vandergeeten_2015 and normalized by reference gene RPP30. Assay Yu_2008 whose amplicon is located in LTR and Gag region of HIV-1 was added as a control ("control assay"). When the DNA is restricted, there is an increase in the measured copies HIV-1 DNA per 10^6 PBMCs by the LTR-assays, but not for the control assay (Fig. S4A). Ratio of HIV-1 DNA measured by LTR-assays versus the control assay is depicted in Figure S4B. However, even when the DNA is not enzymatically restricted, a higher quantification of HIV-1 DNA by the LTR-assays is observed. When the sample is additionally restricted, there is an increase of the ratio observed: more LTR regions of a single HIV-1 DNA genome are separated and incorporated in different droplets. The ratio is higher for HIV-infected SupT1 than for HIV-1 latent ACH2 cell line. HIV-infected SupT1 represent recent infected cells and HIV-1 DNA comprises high level of 2LTR-circles, whereas ACH2 consists of one integrated HIV-1 DNA copy per cell and a low level of 2LTR-circles. Breakage of 2LTR in a random region of the HIV-1 genome will not lead to separation of the LTR and will not lead to an overestimation of the present HIV-1 DNA genomes. Therefore, restriction of DNA of ACH2 with a lower level of 2LTR will lead to a higher

overestimation of HIV-1 DNA levels. The low level of 2LTR explains again that ACH2 cells approximates a ratio of 2: almost all HIV-1 DNA genome are accounted for twice.

HIV-1 DNA quantification in patient samples by qPCR

Until today, qPCR is the most common used PCR platform for HIV-1 DNA quantification. Therefore, the performance of the selected HIV-1 DNA assays was confirmed using qPCR. When available, gDNA was extracted from an additional aliquot of the patient sample, as described before. Total HIV-1 DNA was measured by the 6 best performing assays using qPCR (LightCycler® 480 System, Roche). Data was normalized by measurements of RPP30 by qPCR. Shortly, 2 µl gDNA was added to a 20 µl reaction consisting of 10µl LightCycler® 480 Probes Master, 400 nM primers and 200 nM probe. After a pre-incubation step of 5 min at 95°C followed 45 cycles of amplification for 15 sec at 95°C and 1 min at 60°C and completed by a cooling step of 15 min at 40°C (ramp rate 2.5°C/sec). Level of $\log_{10}$ HIV DNA/10^6 PBMCs was divided by the maximum number measured for that particular sample to obtain a relative value and was visual represented by a heatmap (package pheatmap, R Development Core Team, 2016) (Fig. S5). On average the 6 tested HIV-1 DNA assays were able to pick up 90% of the maximum measured HIV-1 DNA copies (Schvachsa_2007: 90.0%, Viard_2004: 93.3%, Yu_2008: 93.7%, Van_der_Sluis_2013: 88.5%, Yun_2002: 91.4% and Heeregrave_2009: 73.5%).

References

1. Beloukas, A., Paraskevis, D., Haida, C., Sypsa, V. & Hatzakis, A. Development and assessment of a multiplex real-time PCR assay for quantification of human immunodeficiency virus type 1 DNA. *J. Clin. Microbiol.* **47,** 2194–2199 (2009).

2. Besson, G. J., McMahon, D., Maldarelli, F. & Mellors, J. W. Short-Course Raltegravir Intensification Does Not Increase 2 Long Terminal Repeat Episomal HIV-1 DNA in Patients on Effective Antiretroviral Therapy. *Clin. Infect. Dis.* **54,** 451–453 (2012).

3. Bosman, K. J. *et al.* Comparison of digital PCR platforms and semi-nested qPCR as a tool to determine the size of the HIV reservoir. *Sci. Rep.* **5,** 13811 (2015).

4. Candotti, D., Temple, J., Owusu-Ofori, S. & Allain, J.-P. Multiplex real-time quantitative RT-PCR assay for hepatitis B virus, hepatitis C virus, and human immunodeficiency virus type 1. *J. Virol. Methods* **118,** 39–47 (2004).

5. Chomont, N. *et al.* HIV reservoir size and persistence are driven by T cell survival and homeostatic proliferation. *Nat. Med.* **15,** 893–900 (2009).

6. Chun, T.-W. *et al.* HIV-infected individuals receiving effective antiviral therapy for extended periods of time continually replenish their viral reservoir. *J. Clin. Invest.* **115,** 3250 (2005).

7. Claiborne, D. T. *et al.* Replicative fitness of transmitted HIV-1 drives acute immune activation, proviral load in memory CD4+ T cells, and disease progression. *Proc. Natl. Acad. Sci.* **112,** E1480–E1489 (2015).

8. Cochrane, A. *et al.* High levels of human immunodeficiency virus infection of CD8 lymphocytes expressing CD4 in vivo. *J. Virol.* **78,** 9862–9871 (2004).

9. Côrtes, F. H. *et al.* HIV controllers with different viral load cutoff levels have distinct virologic and immunologic profiles. *J. Acquir. Immune Defic. Syndr. 1999* **68,** 377–385 (2015).

10. Delobel, P. *et al.* Naive T-cell depletion related to infection by X4 human immunodeficiency virus type 1 in poor immunological responders to highly active antiretroviral therapy. *J. Virol.* **80,** 10229–10236 (2006).

11. Désiré, N. *et al.* Quantification of human immunodeficiency virus type 1 proviral load by a TaqMan real-time PCR assay. *J. Clin. Microbiol.* **39,** 1303–1310 (2001).

12. Douek, D. C. *et al.* HIV preferentially infects HIV-specific CD4+ T cells. *Nature* **417,** 95–98 (2002).

13. Drosten, C., Müller-Kunert, E., Dietrich, M., Gerdes, J. & Schmitz, H. Topographic and quantitative display of integrated human immunodeficiency virus-1 provirus DNA in human lymph nodes by real-time polymerase chain reaction. *J. Mol. Diagn. JMD* **7,** 219–225 (2005).

14. Durand, C. M. *et al.* HIV-1 DNA is detected in bone marrow populations containing CD4+ T cells but is not found in purified CD34+ hematopoietic progenitor cells in most patients on antiretroviral therapy. *J. Infect. Dis.* **205,** 1014–1018 (2012).

15. Ellis, G. M., Page, L. C., Burman, B. E., Buskin, S. & Frenkel, L. M. Increased detection of HIV-1 drug resistance at time of diagnosis by testing viral DNA with a sensitive assay. *J. Acquir. Immune Defic. Syndr. 1999* **51,** 283–289 (2009).

16. Eriksson, L. E., Leitner, T., Wahren, B., Bostrom, A.-C. & Falk, K. I. A multiplex real-time PCR for quantification of HIV-1 DNA and the human albumin gene in CD4+ cells. *APMIS Acta Pathol. Microbiol. Immunol. Scand.* **111,** 625–633 (2003).

17. Eriksson, S. *et al.* Comparative analysis of measures of viral reservoirs in HIV-1 eradication studies. *PLoS Pathog* **9,** e1003174 (2013).

18. Fabre-Mersseman, V. *et al.* $CD4^+$ recent thymic emigrants are infected by HIV in vivo, implication for pathogenesis. *AIDS Lond. Engl.* **25,** 1153–1162 (2011).

19. Soriano-Sarabia, N. *et al.* Control of HIV-1 RNA load after HAART interruption: Relationship with CCR5 co-receptor density and proviral DNA load in HIV-infected patients. *J. Clin. Virol.* **40,** 64–67 (2007).

20. Gandhi, R. T. *et al.* No effect of raltegravir intensification on viral replication markers in the blood of HIV-1-infected patients receiving antiretroviral therapy. *J. Acquir. Immune Defic. Syndr.* **59,** 229–235 (2012).

21. Gantt, S. *et al.* Laboratory indicators of mastitis are not associated with elevated HIV-1 DNA loads or predictive of HIV-1 RNA loads in breast milk. *J. Infect. Dis.* **196,** 570–576 (2007).

22. Ghosh, M. K. *et al.* Quantitation of human immunodeficiency virus type 1 in breast milk. *J. Clin. Microbiol.* **41,** 2465–2470 (2003).

23. Yu, J. J. *et al.* A more precise HIV integration assay designed to detect small differences finds lower levels of integrated DNA in HAART treated patients. *Virology* **379,** 78–86 (2008).

24. Heeregrave, E. J. *et al.* Lack of in vivo compartmentalization among HIV-1 infected naive and memory CD4+ T cell subsets. *Virology* **393,** 24–32 (2009).

25. Hütter, G. *et al.* Long-term control of HIV by CCR5 Delta32/Delta32 stem-cell transplantation. *N. Engl. J. Med.* **360,** 692–698 (2009).

26. Julg, B. *et al.* Infrequent recovery of HIV from but robust exogenous infection of activated CD4+ T cells in HIV elite controllers. *Clin. Infect. Dis.* **51,** 233–238 (2010).

27. Koelsch, K. K. *et al.* Dynamics of total, linear nonintegrated, and integrated HIV-1 DNA in vivo and in vitro. *J. Infect. Dis.* **197,** 411–419 (2008).

28. Kondo, M. *et al.* Quantitation of HIV-1 group M proviral DNA using TaqMan MGB real-time PCR. *J. Virol. Methods* **157,** 141–146 (2009).

29. Kostrikis, L. G. *et al.* Quantitation of human immunodeficiency virus type 1 DNA forms with the second template switch in peripheral blood cells predicts disease progression independently of plasma RNA load. *J. Virol.* **76,** 10099–10108 (2002).

30. Lam, Y. M. P. *et al.* Switching virally suppressed, treatment-experienced patients to a raltegravir-containing regimen does not alter levels of HIV-1 DNA. *PloS One* **7,** e31990 (2012).

31. Lewin, S. R. *et al.* Virologic determinants of success after structured treatment interruptions of antiretrovirals in acute HIV-1 infection. *J. Acquir. Immune Defic. Syndr.* **47,** 140–147 (2008).

32. Luo, W., Yang, H., Rathbun, K., Pau, C.-P. & Ou, C.-Y. Detection of human immunodeficiency virus type 1 DNA in dried blood spots by a duplex real-time PCR assay. *J. Clin. Microbiol.* **43,** 1851–1857 (2005).

33. MacNeil, A. *et al.* Genomic Sites of Human Immunodeficiency Virus Type 2 (HIV-2) Integration: Similarities to HIV-1 In Vitro and Possible Differences In Vivo. *J. Virol.* **80,** 7316–7321 (2006).

34. Maenetje, P. *et al.* A steady state of CD4+ T cell memory maturation and activation is established during primary subtype C HIV-1 infection. *J. Immunol.* **184,** 4926–4935 (2010).

35. Malnati, M. S. *et al.* A universal real-time PCR assay for the quantification of group-M HIV-1 proviral load. *Nat. Protoc.* **3,** 1240–1248 (2008).

36. Mitsuyasu, R. T. *et al.* Prolonged survival and tissue trafficking following adoptive transfer of CD4ζ gene-modified autologous CD4+ and CD8+ T cells in human immunodeficiency virus–infected subjects. *Blood* **96,** 785–793 (2000).

37. Mohey, R. *et al.* Detection and quantification of proviral HIV-1 184 M/V in circulating CD4(+) T cells of patients on HAART with a viremia less than 1,000 copies/ml. *J. Clin. Virol. Off. Publ. Pan Am. Soc. Clin. Virol.* **34,** 257–267 (2005).

38. Novitsky, V. A. *et al.* Interactive association of proviral load and IFN-γ-secreting T cell responses in HIV-1C infection. *Virology* **349,** 142–155 (2006).

39. Ometto, L. *et al.* Immune reconstitution in HIV-1-infected children on antiretroviral therapy: role of thymic output and viral fitness. *Aids* **16,** 839–849 (2002).

40. Pallikkuth, S. *et al.* Peripheral T Follicular Helper Cells Are the Major HIV Reservoir within Central Memory CD4 T Cells in Peripheral Blood from Chronically HIV-Infected Individuals on Combination Antiretroviral Therapy. *J. Virol.* **90,** 2718–2728 (2015).

41. Pasternak, A. O. *et al.* Highly Sensitive Methods Based on Seminested Real-Time Reverse Transcription-PCR for Quantitation of Human Immunodeficiency Virus Type 1 Unspliced and Multiply Spliced RNA and Proviral DNA. *J. Clin. Microbiol.* **46,** 2206–2211 (2008).

42. Pasternak, A. O. *et al.* Minor Contribution of Chimeric Host-HIV Readthrough Transcripts to the Level of HIV Cell-Associated gag RNA. *J. Virol.* **90,** 1148–1151 (2015).

43. Rousseau, C. M. *et al.* Association of levels of HIV-1—infected breast milk cells and risk of mother-to-child transmission. *J. Infect. Dis.* **190,** 1880–1888 (2004).

44. Schvachsa, N. *et al.* Examination of real-time PCR for HIV-1 RNA and DNA quantitation in patients infected with HIV-1 BF intersubtype recombinant variants. *J. Virol. Methods* **140,** 222–227 (2007).

45. Serrano-Villar, S. *et al.* Effects of Combined CCR5/Integrase Inhibitors-Based Regimen on Mucosal Immunity in HIV-Infected Patients Naïve to Antiretroviral Therapy: A Pilot Randomized Trial. *PLoS Pathog.* **12,** e1005381 (2016).

46. Shete, A. *et al.* Short communication: HIV antigen-specific reactivation of HIV infection from cellular reservoirs: implications in the settings of therapeutic vaccinations. *AIDS Res. Hum. Retroviruses* **28,** 835–843 (2012).

47. Soares, R. *et al.* Increased Frequency of Circulating CCR5+ CD4+ T Cells in Human Immunodeficiency Virus Type 2 Infection. *J. Virol.* **80,** 12425–12429 (2006).

48. Strain, M. C. *et al.* Highly precise measurement of HIV DNA by droplet digital PCR. *PloS One* **8,** e55943 (2013).

49. Torres-Cornejo, A. *et al.* Cellular HIV reservoir replenishment is not affected by blip or intermittent viremia episodes during darunavir/ritonavir monotherapy. *AIDS Lond. Engl.* **28,** 201–208 (2014).

50. van der Sluis, R. M. *et al.* Quantitation of HIV-1 DNA with a sensitive TaqMan assay that has broad subtype specificity. *J. Virol. Methods* **187,** 94–102 (2013).

51. Vandergeeten, C. *et al.* Cross-clade ultrasensitive PCR-based assays to measure HIV persistence in large-cohort studies. *J. Virol.* **88,** 12385–12396 (2014).

52. Viard, J.-P. *et al.* Impact of 5 years of maximally successful highly active antiretroviral therapy on CD4 cell count and HIV-1 DNA level. *Aids* **18,** 45–49 (2004).

53. Wada, K. *et al.* Delayed HIV-1 Infection of CD4+ T Lymphocytes from Therapy-Naïve Patients Demonstrated by Quantification of HIV-1 DNA Copy Numbers. *Microbiol. Immunol.* **48,** 767–772 (2004).

54. Wilkinson, J. *et al.* A Phase 1b/2a study of the safety, pharmacokinetics and antiviral activity of BIT225 in patients with HIV-1 infection. *J. Antimicrob. Chemother.* **71,** 731–738 (2016).

55. Williams, J. P. *et al.* HIV-1 DNA predicts disease progression and post-treatment virological control. *Elife* **3,** e03821 (2014).

56. Yukl, S. A. *et al.* Differences in HIV Burden and Immune Activation within the Gut of HIV-Positive Patients Receiving Suppressive Antiretroviral Therapy. *J. Infect. Dis.* **202,** 1553–1561 (2010).

57. Yun, Z., Fredriksson, E. & Sönnerborg, A. Quantification of human immunodeficiency virus type 1 proviral DNA by the TaqMan real-time PCR assay. *J. Clin. Microbiol.* **40,** 3883–3884 (2002).

58. Zhu, W. *et al.* Rapid turnover of 2-LTR HIV-1 DNA during early stage of highly active antiretroviral therapy. *PloS One* **6,** e21081 (2011).

59. Korber B. *et al.* Numbering positions in HIV relative to HXB2CG. Hum Retroviruses AIDS. **3**:102–11 (1998).

Supplemental Figures

Figure S1 : ***In silico* evaluation of total HIV-1 DNA assays by in-house bioinformatics tool**. A varying number of mismatches was allowed between the HIV-1 sequences and the probe sequence (ranging from 0 to 3). HIV-1 DNA assays selected for further analysis are depicted in blue.

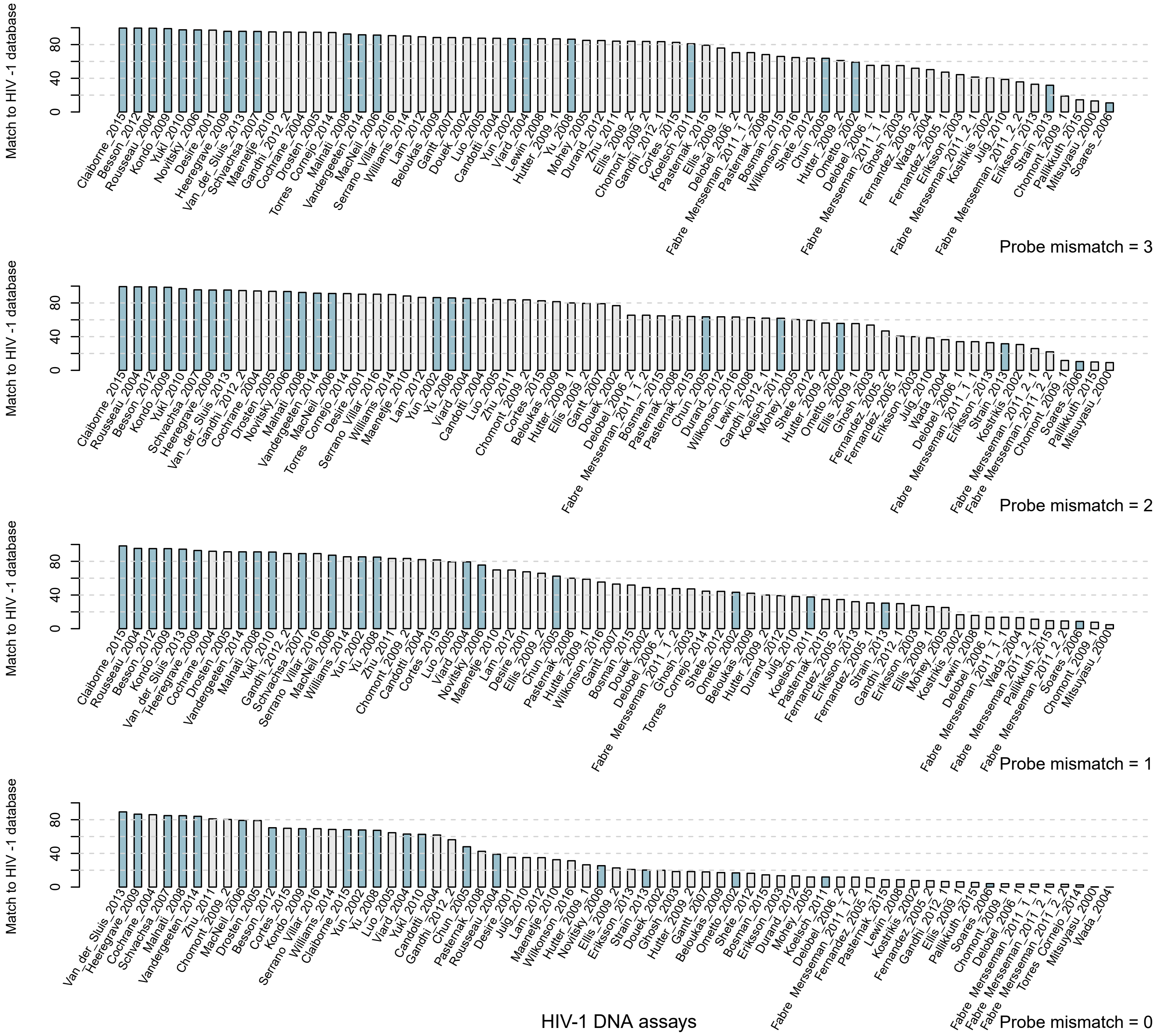

Figure S2: **Analysis of restriction recognition sites in the HIV genome.** Frequency of restriction site situated in the HIV genome is represented by the red line. Arrow (↑) represents a frequency of 100%. HIV-1 gene map based on HXB2 reference gene[59].

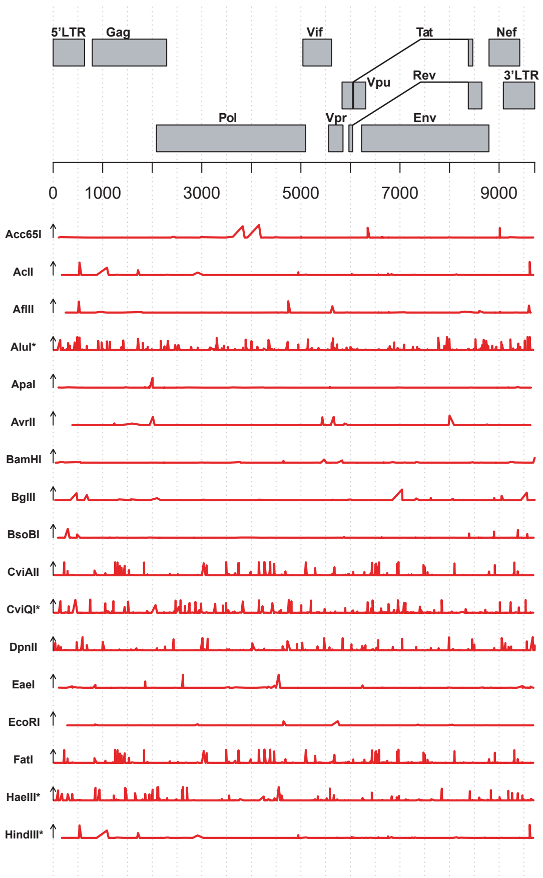

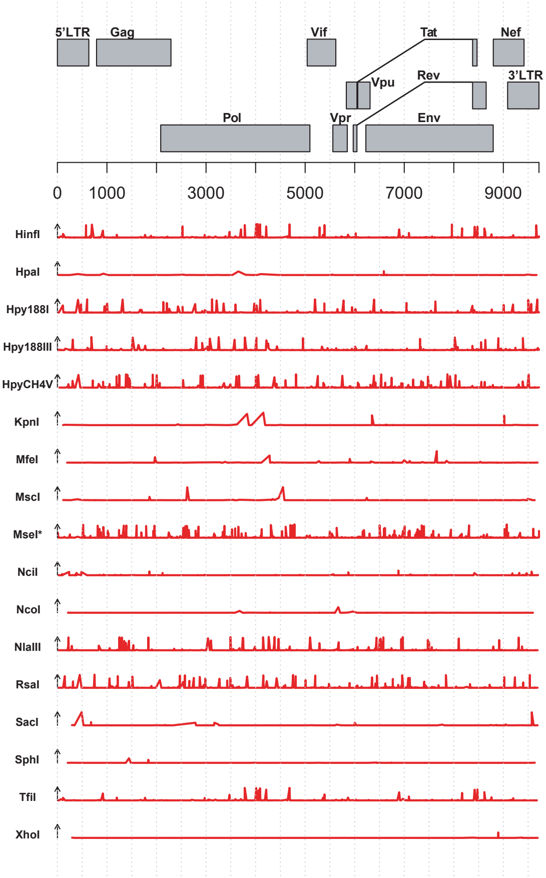

Figure S3: **Restriction recognition site analysis for 20 total HIV-1 DNA assays selected based on in-house bioinformatics tool.** Each square represents a restriction enzyme. Each bar represents the percentage of amplicons for a specific HIV-1 DNA assay that are disrupted based on the HIV Los Alamos database.

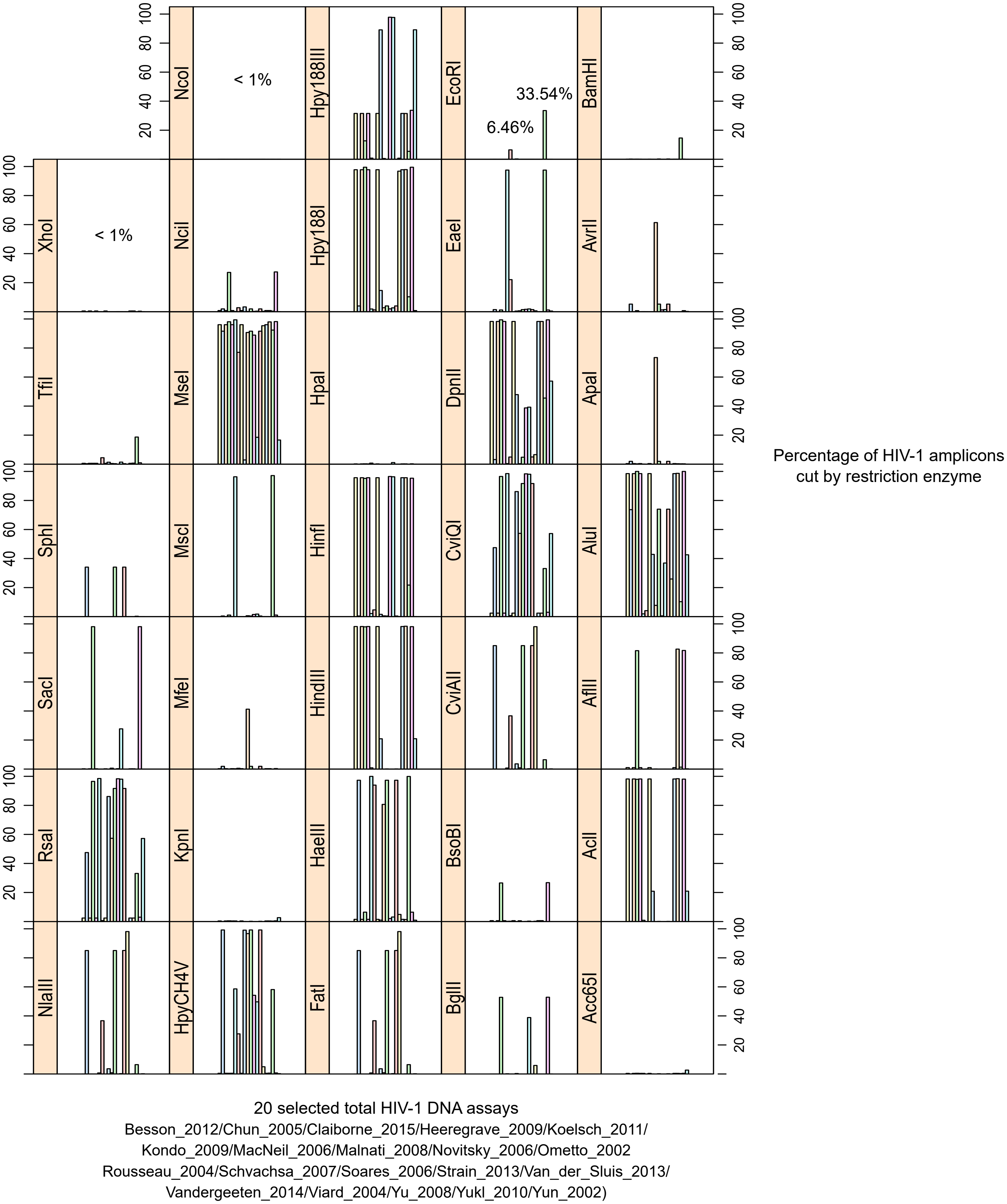

Figure S4: **Assessment of HIV-1 DNA assays located solely in the HIV-1 LTR region (Vandergeeten_2014 and Chun_2005) versus control assay (Yu_2008).** (A.) Quantification of HIV-1 DNA in restricted versus non-restricted gDNA of HIV-1-infected SupT1 and ACH2 cell line. (B.) Ratio of HIV-1 DNA quantification of LTR -assay versus control assay.

A.

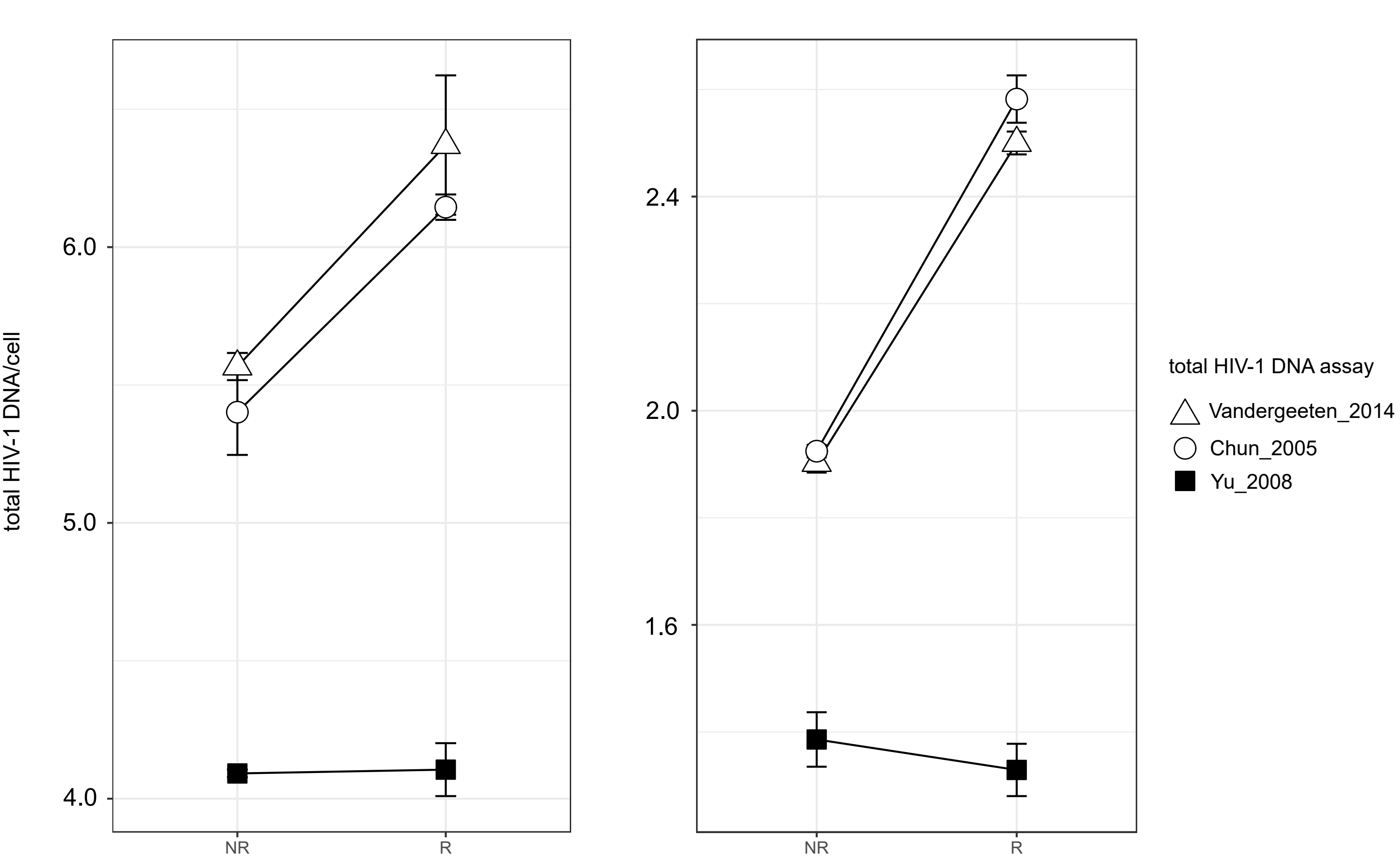

B.

Ratio HIV-1 DNA		Vandergeeten_2014/Yu_2008	Chun_2005/Yu_2008
SupT1	**Restricted**	1.6	1.5
	**Non-restricted**	1.4	1.3
ACH2	**Restricted**	1.9	1.9
	**Non-restricted**	1.4	1.4

Figure S5: **Heatmap of log HIV DNA/10^6 PBMCs measured on the qPCR platform.** Relative HIV-1 DNA quantification results per sample show the assays with high sensitivity (red) and the assays with lower sensitivity (blue).

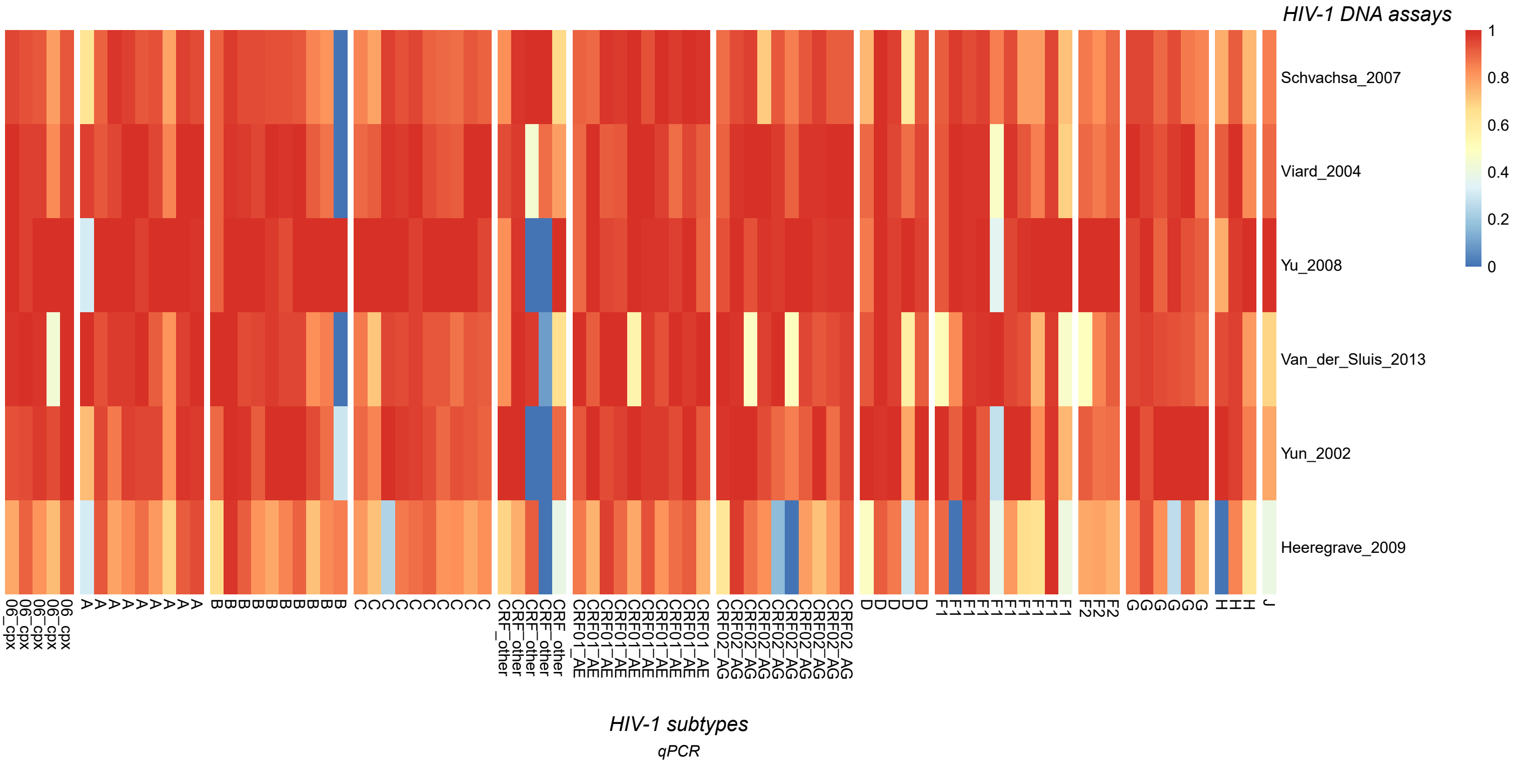

Supplemental Tables

Table S1: Total HIV-1 DNA assays: Sequences of primers and probes and assay-specific annealing temperature for droplet digital PCR (ddPCR) and real-time PCR (qPCR).

Table S2: Total HIV-1 DNA quantification of patients samples by ddPCR. Copies HIV DNA/million PBMCs quantified by 6 different assays by ddPCR.

Table S3: Total HIV-1 DNA quantification of patients samples by qPCR. Copies HIV DNA/million PBMCs quantified by 6 different assays by qPCR.
